# Proteomic profiling of spontaneous myopia in guinea pigs

**DOI:** 10.3389/fmed.2026.1757831

**Published:** 2026-03-19

**Authors:** Hong Liu, Yongli Zhou, Xiaoning Li, Huixin Sun, Yuwen Hsiao, Longbo Wen, Qinglin Xu, Zhao Chen, Weizhong Lan, Zhikuan Yang

**Affiliations:** 1 Changsha Aier Eye Hospital, Aier Eye Hospital Group, Aier Academy of Ophthalmology, Central South University, Changsha, Hunan, China; 2 Aier Institute of Optometry and Vision Science, Changsha, China; 3 Aier School of Optometry and Vision Science, Hubei University of Science and Technology, Xianning, China; 4 Aier Eye Hospital, Jinan University, Guangzhou, Guangdong, China

**Keywords:** complement cascades, experimental myopia, ferroptosis, guinea pig, high myopia, proteomics, spontaneous myopia

## Abstract

**Purpose:**

There is a subset of guinea pigs born with spontaneous myopia that exhibit fundus characteristics similar to those of high myopia. This study aimed to further identify the traits, including proteomic profiling of the retina and plasma, in this strain of guinea pigs.

**Methods:**

Spontaneously myopic guinea pigs (*n* = 11) and hyperopic controls (*n* = 12) were screened from 2-week-old pigmented guinea pigs. Refractive error (RE) was measured using infrared eccentric photorefraction. Swept-source optical coherence tomography (SS-OCT) was employed to assess ocular biometrics in anterior segment mode, while posterior layer thicknesses and fundus images were obtained in posterior segment mode. Retinal activity was assessed using ffERG. H&E staining and TUNEL assay were performed, with the latter assessing apoptosis in the retina and optic disc head. Retinal and plasma samples were further analyzed using rapid data-independent acquisition (Rapid-DIA) proteomics.

**Results:**

Compared to hyperopic eyes, spontaneously myopic eyes displayed a more negative RE (*p* < 0.0001), significantly elongated AL (*p* = 0.004), and increased VCD (*p* < 0.0001). Moreover, spontaneously myopic eyes exhibited significantly thinner retina (*p* = 0.008), choroid (*p* = 0.0006), and sclera (*p* = 0.002). All spontaneous myopia cases displayed a tessellated fundus, whereas no such fundus was observed in hyperopia. ERG responses were similar between the two groups. TUNEL staining revealed significantly increased apoptosis in the outer nuclear layer of myopic retinas. Proteomic analysis identified upregulated complement activation and ferroptosis-related pathways in myopic retinas, alongside reduced nitric oxide signaling protein expression. Plasma proteomics indicated elevated VEGF pathway protein expression in spontaneously myopic guinea pigs.

**Conclusion:**

Spontaneously myopic guinea pigs showed high similarity to human high myopia in ocular parameters, fundus, and molecular pathways, suggesting the potential as an alternative model for high myopia research.

## 1 Introduction

Myopia has emerged as a growing global concern in recent decades, with East Asia being particularly affected. In China, the incidence of myopia among adolescents aged 6 to 17 is 37.4% (1), while among high school students aged 16 to 18, the incidence rises to 84.8% (2). Notably, the prevalence of high myopia in adolescents is as high as 19.3% (2). Although myopic symptoms can be corrected with spectacle lenses, the risks associated with high myopia are substantial, potentially leading to serious complications such as glaucoma and retinal macular disease. Therefore, developing a high myopia model is essential for therapy research.

It was initially reported by Wiesel and Raviola in 1977 that axial myopia could be induced in monkeys through suturing their eyelids (3). Then Wallman established the form deprivation myopia (FDM) model in chickens by attaching occluders on the eyes (4). In 1988, Frank Schaeffel developed lens-induced myopia (LIM) in chickens by imposing hyperopic defocus via negative lenses (5). FDM and LIM have been extensively used in myopia research, and successfully induced in a variety of animals, including chickens, tree shrews, guinea pigs, mice, primates, and even fish [as reviewed by Schaeffel and Feldkaemper (6)]. In addition to the commonly used FDM and LIM, there are alternative animal myopia models induced by other factors. These include myopia resulting from near work (7) and from exposure to monochromatic light in guinea pigs (8). However, these alternative models generally exhibit a lesser degree of myopic shift compared to FDM and LIM.

In 2009, Jiang et al. (9) reported a new type of animal myopia: spontaneously myopic guinea pigs, and described their refractive development. Wild-type pigmented guinea pigs can be born with hyperopia, anisometropia, or myopia. Spontaneous myopia occurs in 70.1% of albino guinea pigs, while the incidence is significantly lower at 28.6% in pigmented guinea pigs (10). Unlike hyperopic guinea pigs, most spontaneously myopic guinea pigs do not develop towards emmetropization. Instead, they exhibit relatively stable negative refractive errors, accompanied by an elongation of the axial length and vitreous chamber depth (9). Nevertheless, the spontaneously myopic guinea pigs can still respond to form deprivation, although the myopic shift induced in spontaneous myopia was less pronounced compared to that in hyperopia (10). Additionally, while retinal dopamine is protective against myopia, its role differs in FDM and spontaneous myopia: it strongly correlates with refraction in FDM but not in spontaneous myopia (11). On the other hand, there are similarities between spontaneous myopia and FDM/LIM. For example, spontaneous myopia is characterized by decreased choroidal thickness and reduced choroidal blood perfusion, similar to FDM and LIM (12). Spontaneously myopic guinea pigs exhibit characteristics closer to pathological myopia, as a high prevalence of fundus tessellation has been observed in this strain (13).

This study was to further elucidate the characteristics of spontaneously myopic guinea pigs. Specifically, the study evaluated fundus morphology, retinal function, and retinal apoptosis. Additionally, retinal and plasma proteomics were performed to identify alterations in protein expression. These investigations are expected to contribute to the assessment of the potential of spontaneously myopic guinea pigs as a high myopia model.

## 2 Methods

### 2.1 Animals

This research was approved by the Ethics Committee at Aier Eye Institute (AEI2025032). Pigmented guinea pigs at age of 2 weeks were purchased from Hunan Taiping Biotechnology Company and raised in the animal facility in Aier Eye Institute. The illuminance was approximately 400 lux, under a daily 12/12 light/dark cycle. The room temperature was maintained at 25 °C. Water and food were provided ad libitum, with fresh vegetables supplied twice a day. All care and treatment for animals were carried out according to the ARVO statement for the Use of Animals in Ophthalmic and Vision Research. Guinea pigs were screened for refraction upon arrival. Those with refractive error (RE) of both eyes below −1.0 D or above +1.0 D were assigned to the spontaneous myopia or hyperopia groups, respectively. Anesthesia was administered during the electroretinography (ERG) and blood collection procedures. Guinea pigs were anesthetized by intramuscular injection with Zoletil 50 (Virbac, France; a 1:1 combination of tiletamine hydrochloride and zolazepam hydrochloride) at a dose of 5 mg/kg, along with xylazine hydrochloride (Shengxin, China) at a dose of 5 mg/kg. After blood collection, anesthetized guinea pigs were euthanized by gradual CO₂ inhalation. The CO₂ replacement rate was maintained at 30 to 70% of the chamber volume per minute, and the CO₂ flow was sustained for at least 1 min after cessation of animal respiration was observed.

### 2.2 Measurements

#### 2.2.1 Refraction

The RE was assessed using an eccentric infrared photorefractor (Stria.tech, Germany) in alert guinea pigs. Cyclopentolate hydrochloride eye drops (Cyclogyl, Belgium) were applied at intervals of 5 min, with a total of 5 times, followed by a 20-min waiting period before refraction. Measurements were repeated 5 times, and the resulting values were averaged to obtain the final RE.

#### 2.2.2 Swept-source optical coherence tomography (SS-OCT)

**Figure 1:**
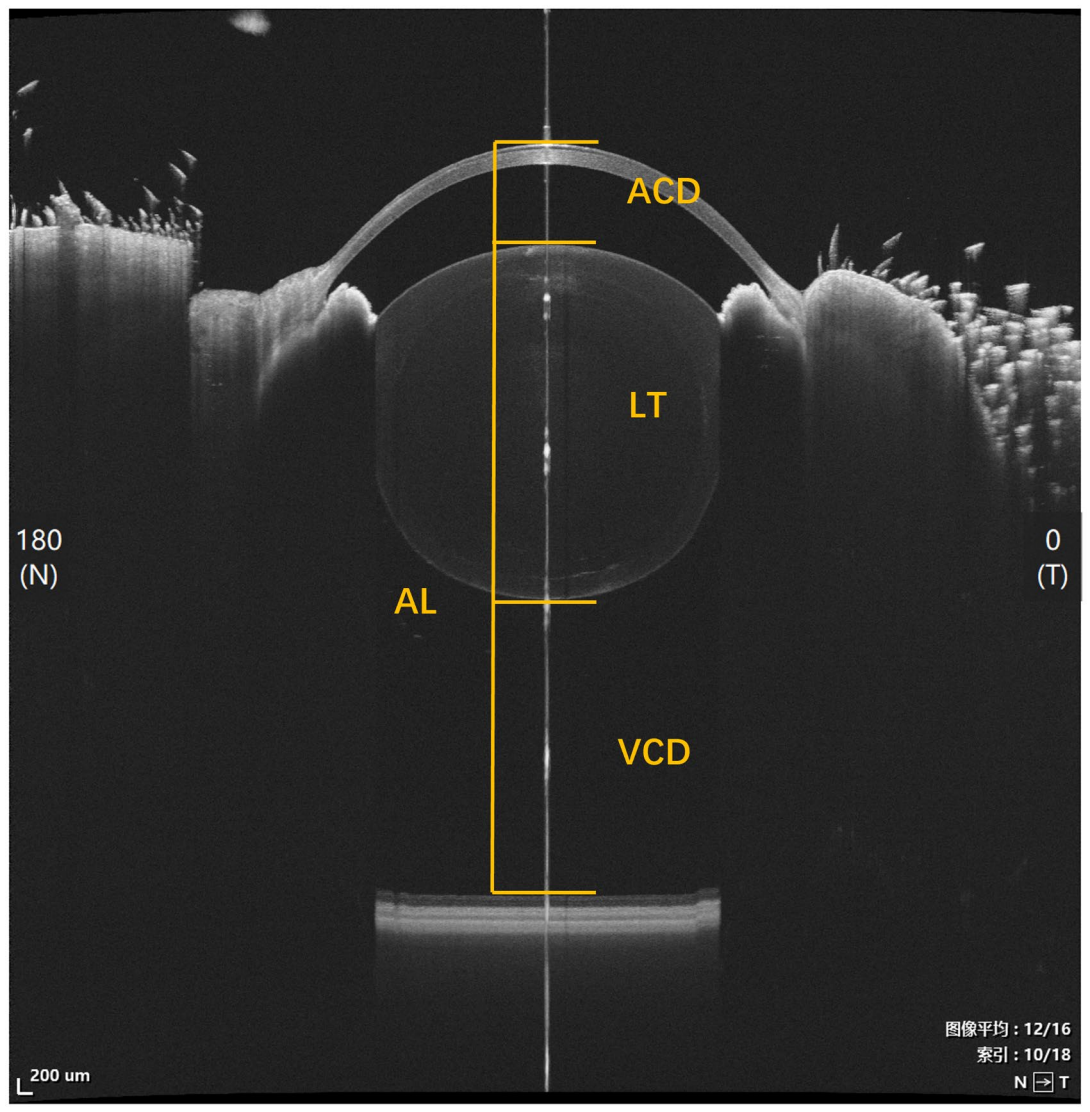
An OCT image taken in anterior segment mode for ocular biometrics. AL, axial length; ACD, anterior chamber depth; LT, lens thickness; VCD, vitreous chamber depth.

Ocular biometrics were measured via SS-OCT (VG200D, Intalight, China). The anterior segment mode of SS-OCT provides a 12 mm depth in tissue, which is sufficient to encompass the entire length of guinea pig eyeballs. All the OCT measurements were taken from 15:00 to 17:00 to minimize the influence of diurnal rhythm effects. Awake guinea pigs were gently held by hand, and their head positions were carefully adjusted to ensure that the cornea was perpendicular to the optical axis of the OCT, with the optical axis passing through the center of the pupil (Figure 1). The length of each ocular part was analyzed with a built-in software of the OCT. Three images were analyzed for each eye.

The posterior layers and fundus image were taken using the posterior segment mode, conducting together with anterior segment-OCT (15:00–17:00). The optical axis was presumed to traverse the center of the pupil, reaching the retina center. For optimal ocular alignment, the optic disc should be positioned in the lower-right quadrant of the fundus image for the right eye, and in the lower-left quadrant for the left eye, as the optic disc is anatomically located in the inferior-temporal region of the fundus in guinea pigs (14). Three images were captured and subsequently analyzed for each eye. The targeted region was at the center of the fundus, situated approximately 700 μm from the optic disc. The thicknesses of the retina, choroid, and sclera were analyzed using ImageJ software, as previously described (15).

#### 2.2.3 Electroretinography (ERG)

Following the measurements of Refraction and OCT, the guinea pigs underwent overnight dark adaptation, after which full-field flash ERG was performed (Q450, Roland Consult, Germany). All the performance were conducted under dim red light. Cyclopentolate hydrochloride eye drops were administered to facilitate pupil dilation and sodium hyaluronate were used to protect cornea. Animals were placed on a 37 ^o^C warming pad after anesthesia to maintain body temperature. Gold wire loop electrodes were placed on the cornea to serve as the active electrode. Needle reference and ground electrodes were, respectively, inserted beneath the skin of the cheek and buttocks. During recording, impedances of ground, reference and two corneal electrodes were maintained below 2 kΩ.

As previously described (16), Scotopic ERGs were performed with white LED stimuli, absent of background lighting, at four intensities (0.01, 3, 10, and 100 cd.s/m^2^). This setup generated rod-dominant responses at lower intensities and mixed responses as intensity increased. For oscillatory potentials (OPs), the low- and high-filter frequency cutoffs were set to 75 Hz and 300 Hz, respectively, to collect the high frequency signals. The rods were subsequently saturated by exposure to white light (25 cd.s/m^2^) for a duration of 10 min, prior to the photopic ERG recordings. An intensity of 3 cd.s/m^2^ was used to detect the cone response. For analysis, the amplitude of the a-wave is defined as the value measured from the baseline to the most prominent negative peak. Similarly, the amplitude of the b-wave is defined as the value from the most prominent negative peak to the most prominent positive peak. The latency is defined as the time interval between the onset of the stimulus and the peak of the response. The OP amplitudes were analyzed using the average value derived from the four OP amplitudes.

#### 2.2.4 Sample preparation and astral LC/MS DIA

Guinea pigs were deeply anesthetized with Zoletile 50 mixed with xylazine hydrochloride as described above, and positioned supinely. A midline incision exposed the heart, from which blood was collected via a sterile needle inserted into the left ventricle. Approximately 1.5 mL blood was extracted into a vacutainer with anticoagulant. The anticoagulant tubes were gently inverted 10 times to ensure thorough mixing of the blood with the anticoagulant, centrifuged immediately at 800 g for 10 min at 4 °C, and then the supernatant plasma was transferred to a 2 mL centrifuge tube. The plasma was subsequently treated with a protease inhibitor and rapidly frozen in liquid nitrogen. The guinea pigs were then euthanized by inhalation of CO₂, and both eyes were carefully enucleated. One eye was designated for proteomic analysis, while the other was reserved for frozen sectioning and staining procedures. The anterior parts of the eyeballs were discarded, and the retinas were dissected and frozen in liquid nitrogen. Both the plasma and the retinas were stored in a −80 °C freezer.

Plasma was mixed with DB buffer (6 M urea, 100 mM TEAB, pH 8.5), centrifuged (4 °C, 12,000 g, 15 min), and the supernatant collected. After adding 1 M DTT, samples were incubated (56 °C, 1 h), cooled on ice, and treated with IAM (dark, RT, 1 h). Retinas were lysed in SDT buffer (100 mM NaCl, DTT), sonicated (ice, 5 min), centrifuged, and the supernatant heated (95 °C, 8–15 min), cooled on ice, and incubated with IAM (dark, 1 h). Samples were mixed with cold acetone, vortexed, and incubated at −20 °C (≥30 min). After centrifugation (12,000 g, 15 min, 4 °C), the pellet was washed with cold acetone and dissolved in DB buffer. Protein concentration was measured (BSA assay), and integrity was checked via SDS-PAGE. Protein samples were digested with trypsin (37 °C, 4 h). After acidification (pH < 3) and centrifugation (12,000 g, 5 min), the supernatant was desalted on a C18 column. Eluents were lyophilized. For the mobile phase, we utilized Phase A, composed of 99.9% water and 0.1% formic acid, and Phase B, which consisted of 80% acetonitrile and 0.1% formic acid.

Mass spectrometry was performed using a Thermo Orbitrap Astral mass spectrometer. The ionization source was an Easy-Spray (ESI), with the ion spray voltage set at 2.0 kV and the ion transfer tube temperature maintained at 290 °C. The mass spectrum acquisition was conducted in a data-independent acquisition (DIA) mode, with a full first-stage mass spectrometry scanning range of 380 to 980 *m/z*. The primary MS resolution was set to 240,000 at *m/z* 200, with an Automatic Gain Control (AGC) target of 500%. The parent ion window size was adjusted to 2 Th, and the number of DIA windows was set to 300. The normalized collision energy (NCE) was set at 25%. The secondary acquisition range extended from 150 to 2,000 *m/z*, with a sub-ion resolution of 80,000. The maximal injection time was limited to 3 milliseconds.

For the analysis of Proteomics, the fixed modification applied is cysteine carbamidomethylation, whereas the excision of the N-terminal methionine (N-term M) is treated as a variable modification. The analysis permits up to two missed cleavage sites. To enhance the accuracy of the analysis, the DIA-NN software employs a filtering process on the search results, retaining only those peptides with a Global Q-Value less than 0.01 and proteins with a PG.Q. Value less than 0.01. The distinction was made by comparing the spontaneous myopia group against the hyperopia group. A protein is identified as a differentially expressed protein (DEP) if it exhibits a fold change (FC) that surpasses 1.2 and *p* < 0.05 (upregulation) or if it drops below 0.83 with a *p*-value less than 0.05 (downregulation). This refined approach ensures that the analysis focuses on high-confidence peptides and proteins, thereby improving the reliability of the differential expression analysis. Gene Ontology (GO) functional analysis were performed using the interproscan program against the non-redundant protein database (17), with COG (Clusters of Orthologous Groups) and KEGG (Kyoto Encyclopedia of Genes and Genomes) databases employed for protein family and pathway analysis. DEPs were utilized for volcano map analysis, and enrichment analysis of GO, and KEGG.

#### 2.2.5 TUNEL and H&E staining

The eyeballs were fixed in FAS fixative solution (G1109, Servicebio, China) immediately after enucleation. To enhance the penetration of fixative solution, two small incisions was made by a 30G needle at the corneal limbus. Following a 24-h fixation period, the anterior segments of the eyes, including the vitreous humor, were removed. Subsequently, the eye cups underwent dehydration using a series of graded glucose solutions (10, 20, 30%), after which they were embedded in OCT freezing medium (Sakura Finetek, Japan, Cat. No. 4583). Subsequently, 10-μm thick sections were made using a cryostat (CM1950, Leica, Germany).

TUNEL (terminal deoxynucleotidyl transferase dUTP nick-end labeling) staining was conducted using a one-step TUNEL cell apoptosis *in situ* detection kit (KeyGen Biotech, Nanjing, China). Following the protocol, frozen sections were air-dried for 1 h, after which they were washed three times in phosphate-buffered saline (PBS; pH 7.4) at room temperature, with each wash lasting 5 min. The sections were then permeabilized in a 1% (v/v) Triton X-100 solution for 5 min, followed by repeated rinsing in PBS as described above. Subsequently, each section was incubated with 40 μL of the TdT enzyme reaction solution, which was prepared by mixing 20 μL of Equilibration Buffer, 10 μL of ddH₂O, 2.0 μL of Biotin-11-dUTP, and 8.0 μL of TdT Enzyme. This incubation was performed in the dark at 37 °C for 1 h, after which the sections were rinsed in PBS three times. Next, 50 μL of the Streptavidin-FITC labeling working solution (5 μL of Streptavidin-FITC mixed with 45 μL of Labeling Buffer) was applied to cover the samples, which were then placed in a humidity chamber and incubated at 37 °C in the dark for 30 min. For nuclear counterstaining, the DAPI staining solution (Servicebio, China, cat. no. G1012) was used, with an incubation period of 10 min at 37 °C in the dark. Finally, the slides were sealed with anti-fluorescence quenching mounting medium and observed under an ortho fluorescence microscope (Axio Scope5, Zeiss, Germany).

For H&E staining, the slides were air-dried at room temperature, followed by three washes in PBS for 5 min. Subsequently, the slides underwent routine H&E staining to facilitate histological assessment. The stained slides were then examined under a light microscope (Primo Vert, Zeiss, Germany). The thickness of the retina, choroid and sclera were quantified at three identical regions (left, middle, and right sides) within each image using ImageJ software. Photoreceptor nuclei in the ONL were quantified. For each eye, three non-consecutive sections were selected, and three fields (100 μm length each) were photographed in the posterior ONL at 40 × magnification. Nuclei were manually counted using ImageJ. Counts from three sections per eye were averaged for each group. Data are presented as nuclei per 100 μm ONL length.

### 2.3 Statistics

Data are shown as the mean ± SEM. The normal distribution of the variables was confirmed via a Shapiro–Wilk normality test. The variations in ocular parameters were analyzed by performing comparisons between the right and left eyes (OD vs. OD, OS vs. OS) utilizing a two-way mixed-repeated measures ANOVA, with eyes as within-subject and group as between-subject factor. A Sidak’s multiple comparisons test was carried out as a *post-hoc* analysis. Given the high degree of interocular homogeneity in ocular parameters observed in both groups, the eye exhibiting a less disrupted waveform was selected for further ERG analysis. Unpaired t-tests were conducted to compare ocular parameters and photopic ERG between groups. Scotopic ERG differences were analyzed using a two-way mixed-repeated measures ANOVA and Sidak’s multiple comparisons, with flash intensity as within-subject and group as between-subject factors.

## 3 Results

### 3.1 Spontaneously myopic eyes have deeper VCD and longer AL

**Figure 2:**
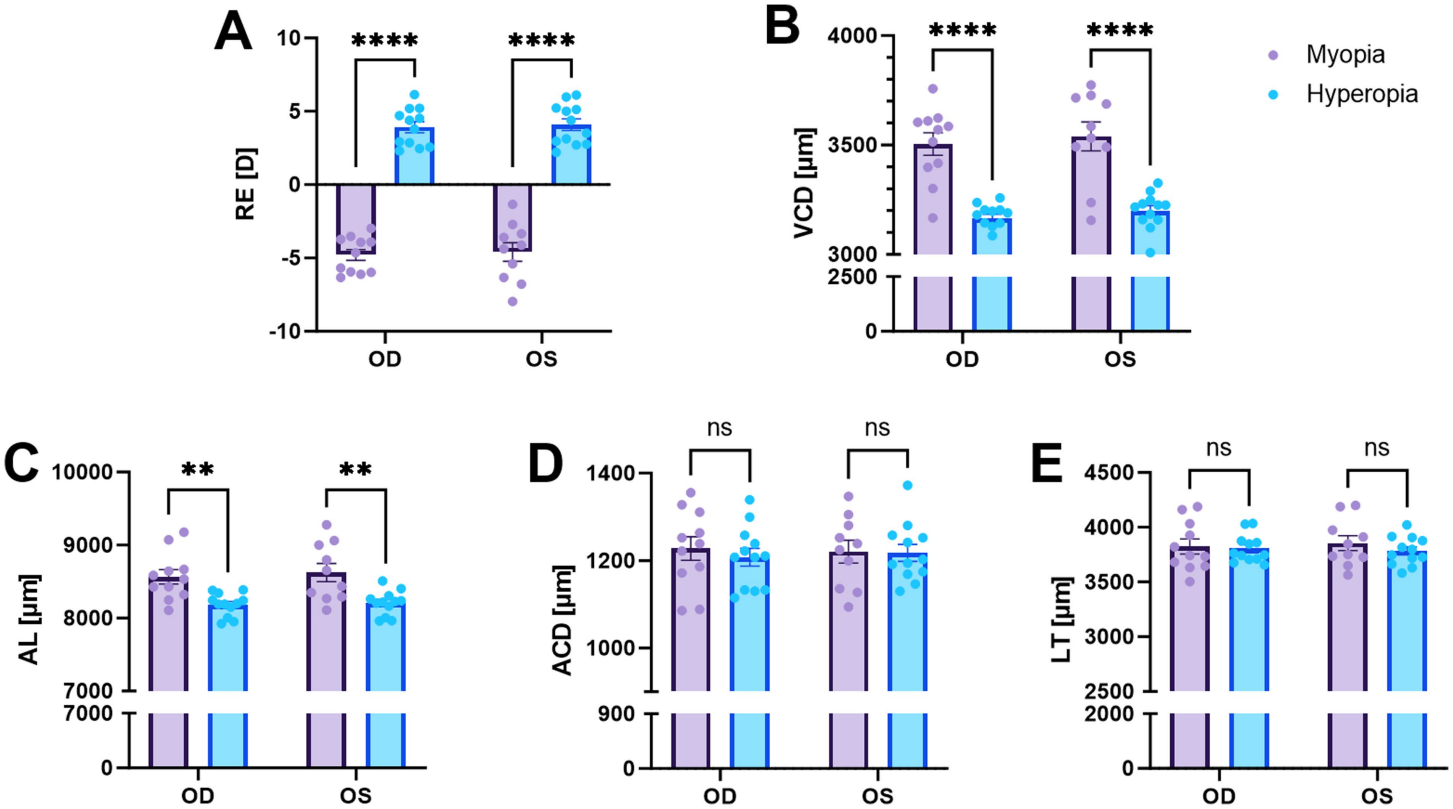
Comparisons of refractive error (RE) and ocular biometrics between spontaneous myopia (purple bars) and hyperopia (blue bars). Compared to hyperopic eyes, spontaneous myopic eyes showed significantly more negative RE **(A)**, deeper VCD **(B)**, and longer AL **(C)**, while ACD **(D)** and LT **(E)** were similar. The right eyes and left eyes were compared between the two groups independently. **p < 0.01; **** *p* < 0.0001; ns: not significant.

The spontaneously myopic guinea pigs showed typical characteristics in RE and ocular biometrics, including myopic RE and extended VCD, whereas their ACD and lens thickness were similar to hyperopic eyes (Figure 2). The right and left eyes within both groups exhibit high homogeneity. Consequently, we conduct a comparative analysis by pairing the eyes separately, that is, right eyes with right eyes and left eyes with left eyes. The RE in two groups are distinctly myopic and hyperopic (*p* < 0.0001, Figure 2A). In consistence with RE, the spontaneously myopic eyes had deeper VCD (both *p* < 0.0001 in right eyes and in left eyes, Figure 2B) and longer AL (*p* = 0.004 in right eyes and *p* = 0.005 in left eyes, Figure 2C). However, no significant disparities were noted in the ACD and LT when comparing the two groups (Figures 2D,E). The detailed data and statistical significance are provided in Table 1.

**Table 1:** Data for RE, ocular biometrics and posterior layer thicknesses of the two strain of guinea pigs.

Parameter	Spontaneous myopia	Hyperopia	*p*-value
RE (D)			
OD	−4.79 ± 0.37	3.92 ± 0.37	<0.0001
OS	−4.59 ± 0.40	4.01 ± 0.40	<0.0001
VCD (μm)			
OD	3,504 ± 51	3,164 ± 21	<0.0001
OS	3,540 ± 61	3,200 ± 24	<0.0001
AL (μm)			
OD	8,565 ± 98	8,184 ± 45	0.004
OS	8,625 ± 125	8,205 ± 48	0.005
ACD (μm)			
OD	1,227 ± 27	1,208 ± 20	0.79
OS	1,220 ± 26	1,218 ± 19	0.99
LT (μm)			
OD	3,824 ± 68	3,804 ± 37	0.95
OS	3,855 ± 68	3,783 ± 38	0.68
Retinal thickness (μm)			
OD	129.4 ± 2.8	138.0 ± 1.0	0.008
OS	128.2 ± 2.6	137.7 ± 1.4	0.004
ChT (μm)			
OD	91.5 ± 9.8	129.3 ± 5.8	0.0006
OS	87.1 ± 4.6	128.2 ± 6.0	0.0002
Scleral thickness (μm)			
OD	84.2 ± 6.4	99.6 ± 3.2	0.07
OS	76.4 ± 6.1	101.6 ± 4.3	0.002

### 3.2 Spontaneous myopia exhibited typical high myopia characteristics in fundus image and posterior layers

**Figure 3:**
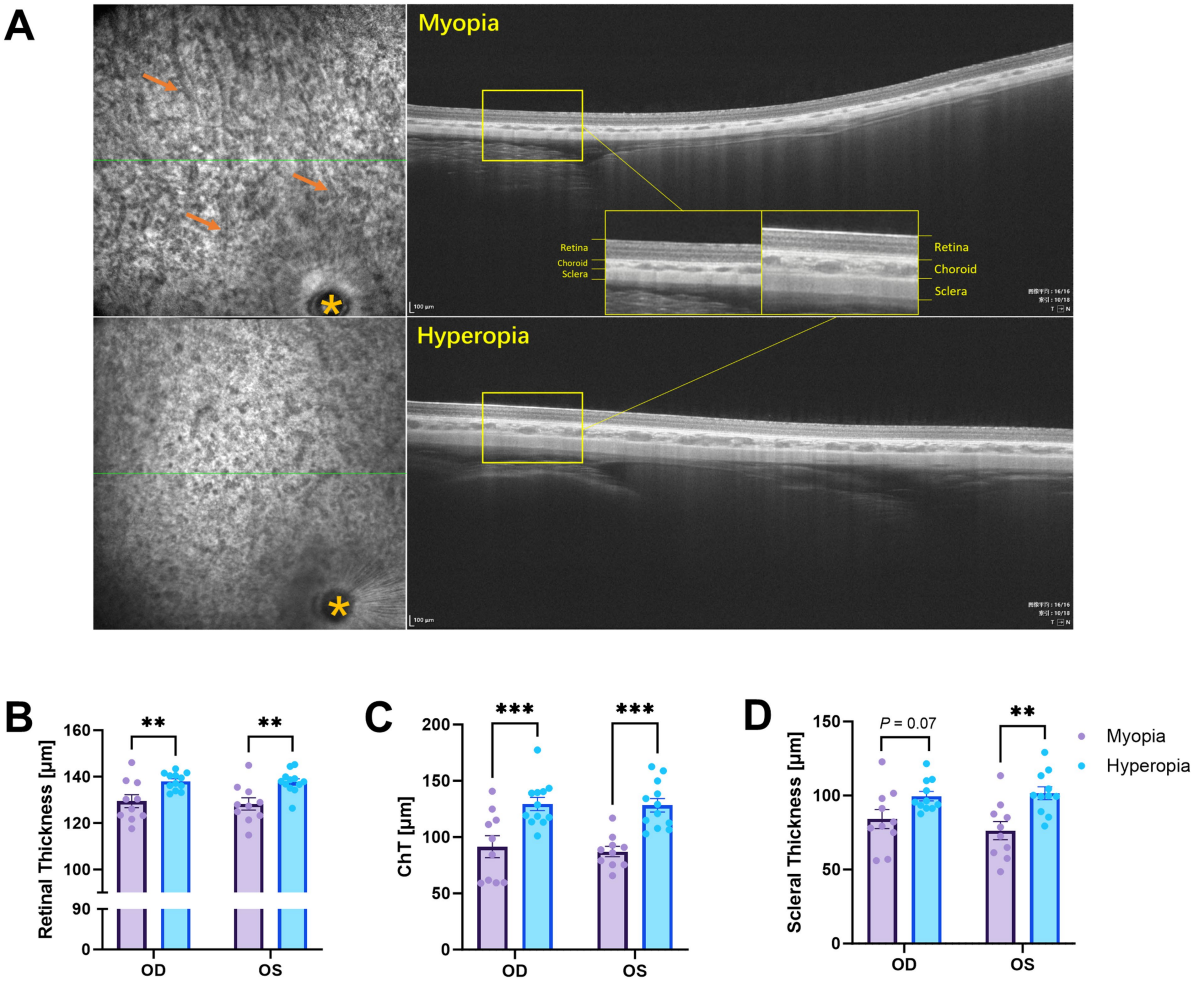
Fundus image and posterior layer thickness in the two groups of guinea pigs. **(A)** OCT images of the right eyes, with the optic disc located in the inferior-temporal area (orange asterisk). Fundus tessellation was observed in spontaneous myopia, characterized by visibly larger blood vessels [orange arrows, **(A)**]. The retinal thickness **(B)**, choroidal thickness [ChT, **(C)**], and scleral thickness **(D)** were all significantly reduced in spontaneous myopia. **p < 0.01; ***p < 0.001; ****p < 0.0001.

The fundus characteristics of spontaneous myopia were similar to those of typical high myopia. The larger blood vessels were clearly visible in the fundus images of the spontaneously myopic eyes, as shown in Figure 3A. Notably, fundus tessellation was observed in 10 out of 10 eyes in the spontaneous myopia group, whereas no such findings were observed in the hyperopia group. The both eyes of spontaneous myopia guinea pigs showed significantly thinner retina (*p* = 0.008 in right eyes, *p* = 0.004 in left eyes, Figure 3B), choroid (*p* = 0.0006 in right eyes, *p* = 0.0002 in left eyes, Figure 3C), and sclera (*p* = 0.07 in right eyes, *p* = 0.002 in left eyes, Figure 3D). The absence of statistical significance in sclera thickness measurements for right eyes was attributed to the presence of a thicker sclera in a single guinea pig. However, this particular measurement was not identified as an outlier. The detailed data and statistical significance are provided in Table 1.

### 3.3 Retinal activity was not affected in spontaneous myopia

**Figure 4:**
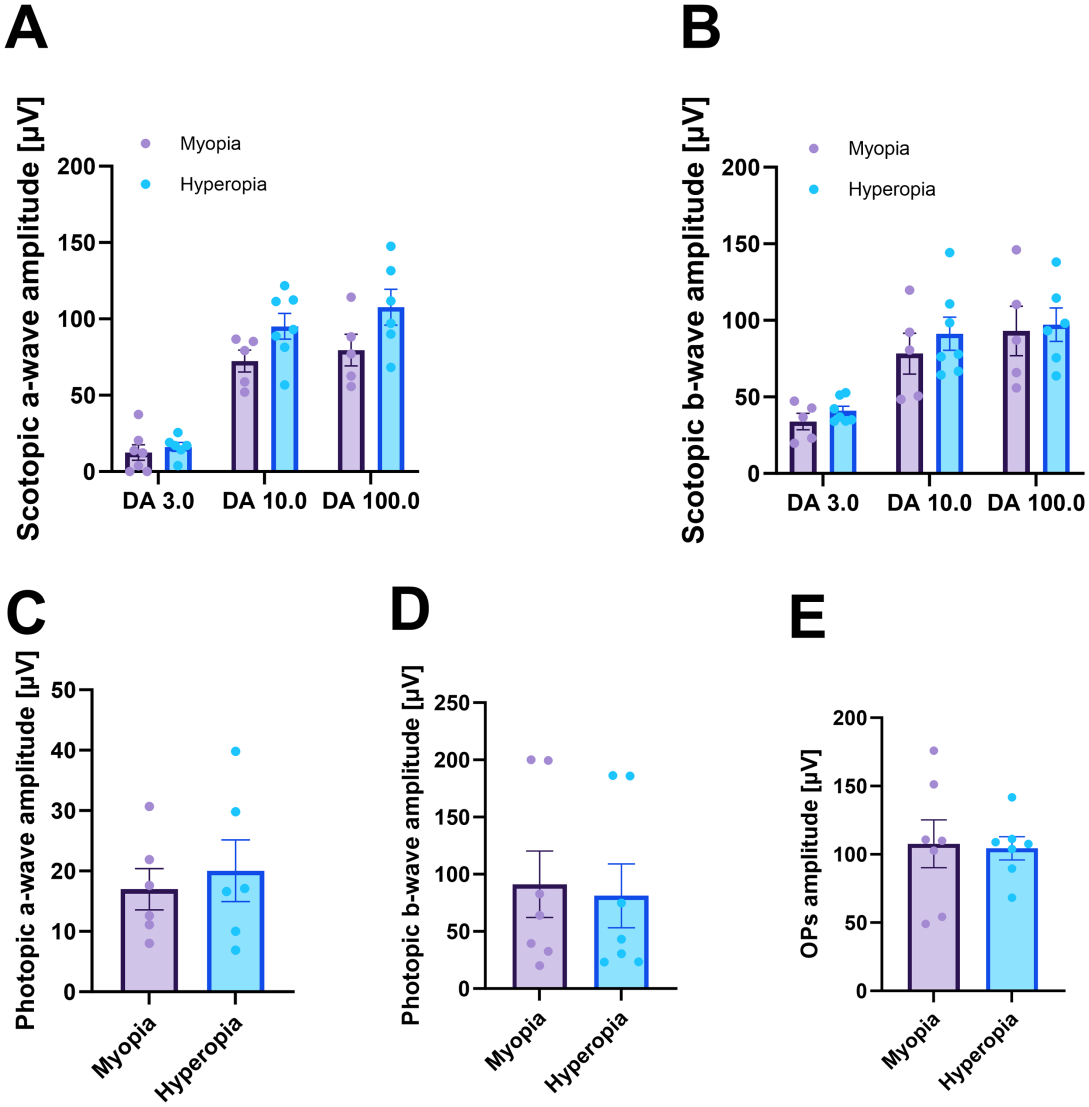
ERG amplitudes in spontaneously myopic and hyperopic guinea pigs. Spontaneous myopia exhibited ERG amplitudes comparable to those of hyperopia, including scotopic a-wave **(A)** and b-wave **(B)**, photopic a-wave **(C)** and b-wave **(D)**, and Ops amplitudes **(E)**.

To evaluate potential changes in retinal function, we conducted ERG in spontaneous myopic and hyperopic guinea pigs. Surprisingly, the retinal activity was similar between the spontaneous myopia and hyperopia. Lower peaks in scotopic a-wave amplitude were observed with increasing flash intensity compared to hyperopic eyes, particularly under 100.0 cd.s/m^2^. However, this difference was not statistically significant (myopia vs. hyperopia under DA 100.0 cd.s/m^2^: 79.6 ± 10.4 μV vs. 107.7 ± 11.8 μV, *p* = 0.07, Figure 4A). Moreover, the scotopic b-wave amplitude, as well as both a- and b-wave amplitudes under photopic conditions, were similar between spontaneously myopic and hyperopic eyes (Figures 4B–D), along with the Ops amplitude (Figure 4E). Additionally, no significant difference in implicit time across all stimuli was found between the two groups (data not shown).

### 3.4 Active apoptosis was observed in the outer nuclear layer, but not in the optic nerve head of spontaneously myopic guinea pigs

**Figure 5:**
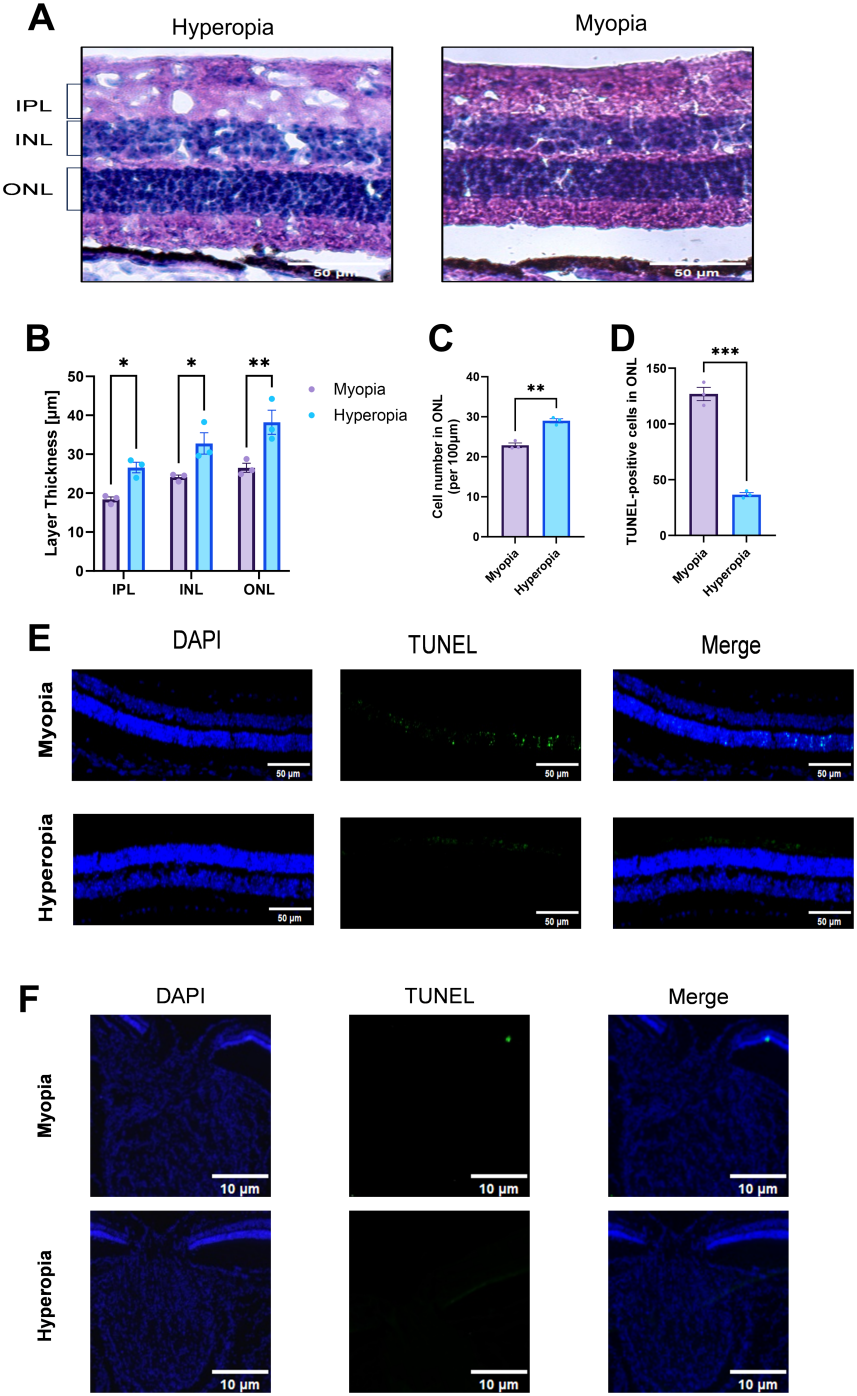
H&E staining and TUNEL immunofluorescence of retinal sections revealed that spontaneously myopic eyes had reduced thickness in the IPL, INL, and ONL **(A,B)**, as well as decreased photoreceptor number in ONL **(C)**. TUNEL staining showed increased apoptosis in the ONL **(D,E)** but no significant activity in the optic nerve head in spontaneously myopic guinea pigs **(F)**. IPL, inner plexiform layer; INL, inner nuclear layer; ONL, outer nuclear layer. *p < 0.05; **p < 0.01; ***p < 0.001.

Further confirmation was provided by HE-stained sections (Figure 5A), revealing retinal thinning in spontaneously myopic guinea pigs. More specifically, the thickness of inner plexiform layer (IPL), inner nuclear layer (INL), and outer nuclear layer (ONL) were all decreased: 18.39 ± 1.11 μm vs. 26.58 ± 2.34 μm in IPL (*p* = 0.03), 24.09 ± 0.95 μm vs. 32.78 ± 4.77 μm in INL (*p* = 0.02), 26.51 ± 2.01 μm vs. 38.19 ± 5.37 μm in ONL (*p* = 0.003), as shown in Figure 5B. Quantitative analysis revealed that photoreceptor number decreased in the thinned ONL. In the hyperopic group, the mean number of photoreceptor nuclei per 100 μm ONL length was 28.98 ± 0.93. In spontaneously myopic group, this value was significantly reduced to 22.88 ± 1.01 (*p* = 0.002, Figure 5C). TUNEL immunofluorescence revealed a significantly higher number of apoptosis-positive cells in the outer nuclear layer (ONL) of spontaneous myopia compared to hyperopia (127 ± 6 vs. 37 ± 2, *p* = 0.0001, Figures 5D,E). However, there was no significant apoptosis detected in the optic nerve head in spontaneous myopia, as shown in Figure 5F.

### 3.5 Proteomics revealed shared signaling pathway between spontaneous myopia and high myopia

**Figure 6:**
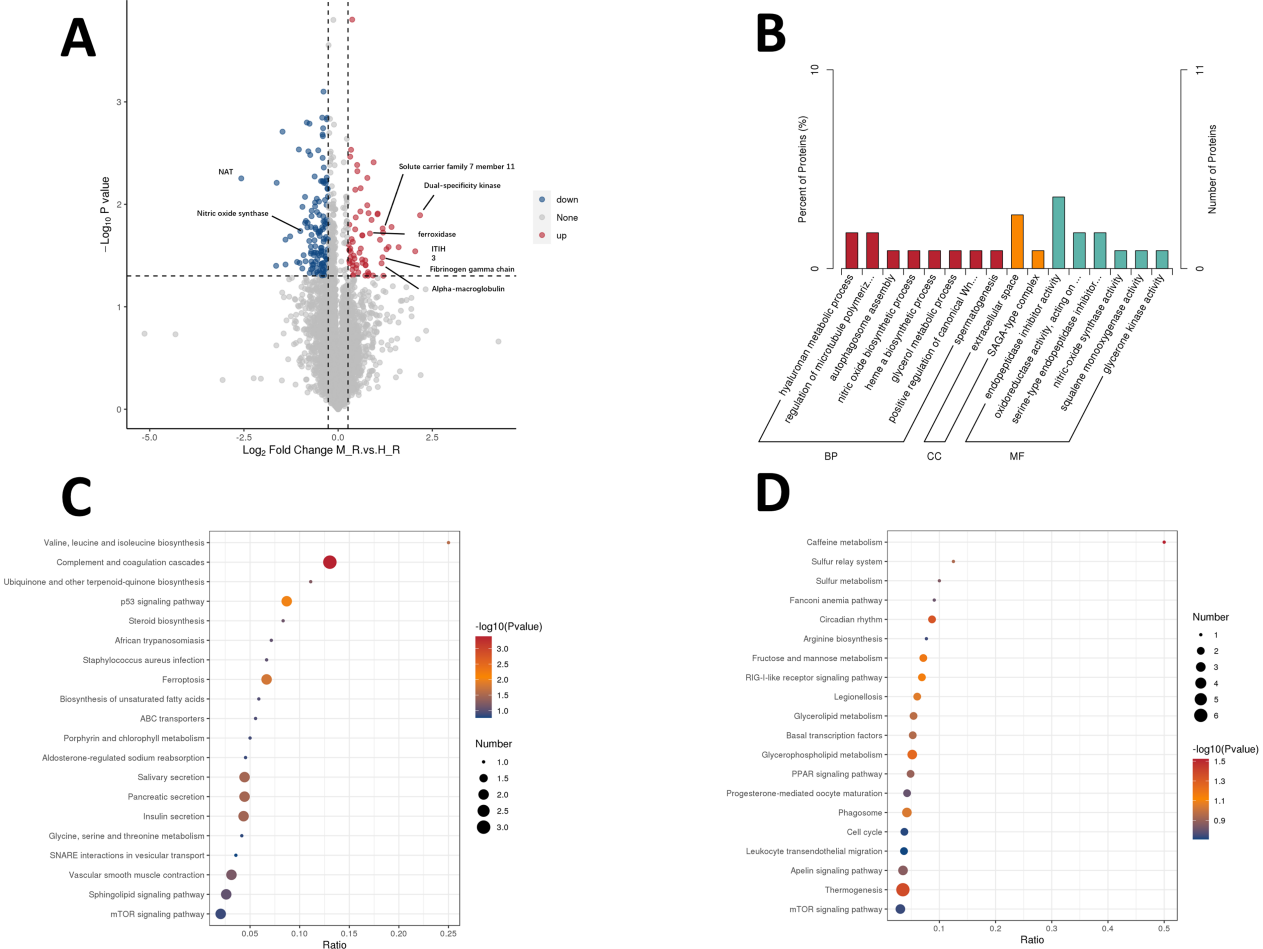
Proteomic profiling of the retina. Volcano plot shows 202 DEPs between spontaneous myopia and hyperopia retinas [67 up, 135 down, **(A)**]. GO analysis revealed enrichment in hyaluronan metabolism, extracellular space, and endopeptidase inhibition **(B)**. KEGG analysis highlighted upregulated pathways valine, leucine, and isoleucine biosynthesis pathway, and the complement and coagulation cascades pathway in spontaneous myopic retina **(C)**. Downregulated DEPs were linked to caffeine metabolism **(D)**. DEPs, differentially expressed proteins.

In the proteomic profiling, a total of 88,071 peptides and 7,869 proteins were identified in the retina of eight samples by DIA. There were 202 differentially expressed proteins (DEPs) identified in the spontaneously myopic retinas compared to the hyperopic retinas, of which 67 DEPs were upregulated and 135 DEPs were downregulated, as illustrated in the volcano plot (Figure 6A). The 3 most upregulated DEPs are dual-specificity kinase (log2 fold change = 2.18), inter-alpha-trypsin inhibitor heavy chain H3 (ITIH3, log2 fold change = 2.05) and inter-alpha-trypsin inhibitor heavy chain H4 (ITIH4, log2 fold change = 1.61), whilst the 3 most downregulated DEPs are arylamine N-acetyltransferase (NAT, log2 fold change = −2.57), Purkinje cell protein 4 like 1 (PCP4L1, log2 fold change = −1.65) and Golgi associated-gamma adaptin ear containing-ARF binding protein 2 (GGA2, log2 fold change = −1.63), in the retina. The functions of the DEPs were further characterized with Gene Ontology (GO) and Kyoto Encyclopedia of Genes and Genomes (KEGG) pathway analyses. Go enrichment analysis revealed that the most significant biological processes (BP) were hyaluronan metabolic process and regulation of microtubule polymerization or depolymerization. The most enriched cellular component (CC) was extracellular space, while the most significant molecular function (MF) was endopeptidase inhibitor activity (Figure 6B). KEGG enrichment analysis highlighted increased expression of pathways associated with valine, leucine, and isoleucine biosynthesis pathway, as well as the complement and coagulation cascades pathway in spontaneous myopic retina (Figure 6C). Meanwhile, the caffeine metabolism pathway was the most significant pathway associated with downregulated DEPs (Figure 6D). The most announced DEPs in the retina are listed in Supplementary Table S1.

**Figure 7:**
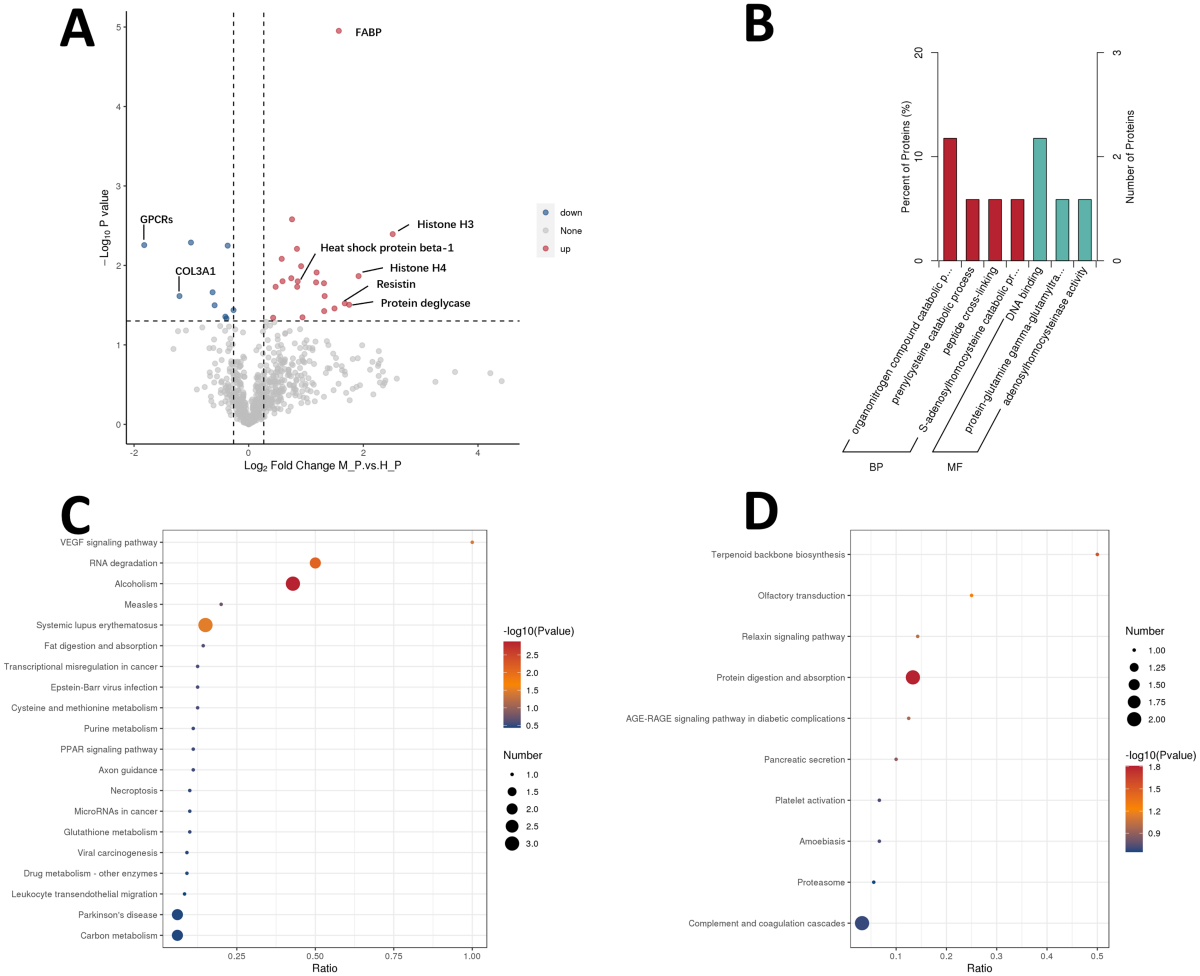
Proteomic profiling of plasma. Volcano plot shows 31 DEPs between spontaneous myopia and hyperopia plasma [22 up, 9 down, **(A)**]. GO analysis highlights organonitrogen catabolism and DNA binding in spontaneously myopic guinea pigs **(B)**. KEGG reveals upregulated pathways in VEGF signaling and RNA degradation **(C)**, while downregulated pathways including terpenoid biosynthesis and olfactory signaling **(D)**. DEPs, differentially expressed proteins.

In contrast, the plasma analysis yielded the identification of 6,689 peptides and 714 proteins, a notably lower count compared to the retina. Among the identified proteins, 31 DEPs were observed, with 22 exhibiting upregulation and 9 showing downregulation (Figure 7A). Among the DEPs, the top 5 most upregulated are: Histone H3 (log2 fold change = 2.51), Histone H4 (log2 fold change = 1.92), protein deglycase (log2 fold change = 1.75), Resistin (log2 fold change = 1.68), and fatty acid-binding protein (FABP, log2 fold change = 1.57). In contrast, the 2 most downregulated DEPs are G-protein coupled receptors family (GPCRs, log2 fold change = −1.82) and collagen type III alpha 1 chain (COL3A1, log2 fold change = −1.21). GO enrichment analysis identified that the most prominent biological process was the organonitrogen compound catabolic process, while the most significant molecular function was DNA binding (Figure 7B). No significant cellular component was identified, a situation likely attributable to the relatively low count of proteins analyzed. KEGG pathway analyses indicated that the most significantly upregulated pathway in spontaneously myopic eyes was VEGF signaling pathway, followed by RNA degradation and alcoholism pathways (Figure 7C). Conversely, the most significantly decreasing expression pathway was terpenoid backbone biosynthesis, followed by olfactory transduction and relaxin signaling pathways (Figure 7D). The most announced DEPs in the plasma are listed in Supplementary Table S2.

## 4 Discussion

In this study, we described the characteristics of the fundus morphology and retinal function of spontaneously myopic guinea pigs, along with the proteomic profiling of the retina and plasma. Spontaneously myopic guinea pigs showed typical signs of high myopia, including elongated eyeball, thinning retina, choroid and sclera, and tessellated fundus. Moreover, proteomics revealed ferroptosis pathway and complement and coagulation cascades were upregulated in spontaneously myopic retina, while nitric oxide signaling protein expression was downregulated. Our study provided additional information on spontaneously myopic guinea pigs, facilitating the evaluation of their potential as an animal model for high myopia.

Expectedly, spontaneously myopic guinea pigs exhibited typical axial myopic ocular parameters, characterized by negative RE, deeper VCD, elongated AL, and unchanged anterior segment length. Unlike experimental mice, there is no genetically uniform strain of pigmented guinea pigs. Consequently, the degree of induced myopia in guinea pigs across different laboratories varies within a certain range. When compared to FDM guinea pigs in our own laboratory, the degree of spontaneous myopia was significantly greater than that induced by 2 weeks of deprivation (16), and was comparable to the myopia resulting from 15 weeks of deprivation (18). Consistent with previous studies, reduced ChT (12) and fundus tessellation (13) were also observed in cases of spontaneous myopia. Employing SS-OCT, we additionally noted decreased retinal and scleral thicknesses in spontaneous myopia. We lack a method such as MRI to assess the presence of staphyloma. Nonetheless, following enucleation, no significant staphyloma was observed from the eyeballs’ morphology.

Fundus tessellation was observed in all spontaneously myopic eyes of this study. Spontaneous myopia has also been documented in macaques. In a prior study, the prevalence of spontaneous high myopia was reported to be 17.7% (19). Fundus tessellation was observed in 78.0% of macaques with high myopia, compared to 37.4% in non-high myopia. In clinic, tessellated fundus represents the first stage of myopic maculopathy and serves as the earliest detectable sign of pathological myopia-related retinal changes (category 1) (20). In a 4-year follow-up clinical trial, 18.2% of adolescents with high myopia developed new-onset fundus tessellation, suggesting a potential precursor to atrophic lesions (21). More severe fundus tessellation associates with lower contrast sensitivity in patients (22).

In this study, increased apoptosis was observed in the ONL of spontaneously myopic retinas, suggesting photoreceptor degeneration. Similarly, in green light-induced myopic mice, abundant apoptotic cells were detected in the ONL and INL (23). Extensive researches have proved that photoreceptors—both cones and rods—play a pivotal role in the pathogenesis of high myopia. In patients with high myopia, the cone photoreceptor density and spatial regularity were significantly reduced compared to those with emmetropia or low myopia (24). Additionally, structural alterations were observed, including thinning of the myoid and ellipsoid zones, as well as the outer nuclear layer. Furthermore, macular light sensitivity was also diminished in high myopia relative to the control groups (25). The less cone density, larger cone spacing and lower cone regularity were associated with impaired contrast sensitivity in patients with high myopia (26). In addition to cones, rods have also been found to significantly contribute to myopia. In monkeys, peripheral FDM could still be induced following laser ablation of the cone-dominant fovea, with a severity comparable to that in intact eyes (27). Meanwhile, the rod-rich peripheral retina exhibited greater sensitivity to retinal defocus than the central retina. Consequently, eye growth rate can be retarded by imposing myopic defocus on the peripheral retina (28). In nocturnal animals such as mice, where photoreceptors are rod-dominated, the role of rods in myopia is particularly critical. Gnat1^−/−^ mice, which lack functional rods, exhibited disrupted emmetropization and absence of responses to form deprivation (29). The underlying mechanism may involve the relationship between rods and dopamine, as a study has shown that dopamine release is strictly rod-dependent, with neither cones nor ipRGCs contributing to this neuromodulatory process in the mouse retina (30). Research findings on ERG changes in myopic eyes are inconsistent. High myopes typically display reduced a-wave and b-wave amplitudes in full-field ERG, and multifocal ERG (mfERG) reveals that the reductions are localized both in the central and peripheral retina (31). However, when assessed using the RETeval system, which employs skin electrodes, the reduced amplitudes typically reported in myopic eyes were not observed (32). In our study, the retinal responses remained similar between spontaneous myopia and hyperopia. In the mouse LIM eyes, both the a-wave and b-wave amplitudes under all stimulating conditions remained similar to those in the control eyes (33). Interestingly, albino guinea pigs—a strain that is more susceptible to experimental myopia—consistently exhibited higher a-wave and b-wave amplitudes than pigmented groups, both before and after form deprivation (34). The authors suggested that pigmentation deficiency in albinos might reduce ocular light absorption, thus enhanced photoreceptor responses. It appears paradoxical that the full-field ERG remained unaffected despite increased apoptosis and a reduced cell count in the ONL observed in our study. This discrepancy may be explained by the following considerations. First, the retinal sections used for TUNEL and H&E staining were obtained from the posterior region near the optic disc. It is possible that the full-field ERG, which reflects global retinal activity, averaged out localized electrophysiological alterations that might be detectable only through multifocal ERG (mfERG). A previous study involving children with progressive myopia reported decreased retinal function in the central retina (35). In comparison, after 6 months of repeated low-level red-light therapy in myopic children, the response density and amplitude of the P1 wave in mfERG were found to be increased specifically in the central foveal region (36). Meanwhile, as relative peripheral refraction (RPR) is critical for myopia progression, a study found that corresponding RPR is not associated with peripheral mfERG signals (37). Second, a study using slow flash-mfERG reported that myopic individuals exhibit reduced P1 and N2 amplitudes compared to emmetropes, whereas N1 amplitudes remain comparable (38). In the first-order waveform, P1 is influenced by both ON- and OFF-bipolar cell activity, N2 originates from the inner retina (amacrine and ganglion cells), and N1 primarily reflects photoreceptor function. Taken together with our findings, these observations suggest that in myopia, electrophysiological alterations in the inner retina—particularly in bipolar cells—may be more pronounced or detectable earlier than those occurring at the photoreceptor level in the ONL.

Proteomic analyses of the retina and plasma were conducted in this study, providing, to our knowledge, the first biomechanical characterization of spontaneously myopic guinea pigs. Ferroxidase expression was elevated in the retina of spontaneous myopia, correlating with ferroptosis in KEGG pathway analysis and enhanced oxidoreductase activity in GO molecular function (GO-MF) analysis. Recent studies have revealed that ferroptosis contributes significantly to myopia pathogenesis. Elevated ferroptosis was detected in the sclera of form-deprived guinea pigs, and pharmacological inhibition of ferroptosis by quercetin (39) and glycine supplementation (40) effectively attenuated myopia progression. Moreover, ferroptosis was significantly upregulated in the retinas of myopic mice (41) and contributed to the formation of highly myopic cataract (42). Among the enriched signaling pathways identified in the KEGG analysis, the upregulation of complement and coagulation cascades in the spontaneous myopia retina drew our attention, with fibrinogen gamma chain and alpha-macroglobulin emerging as a key DEPs. This finding aligns with a previous study, which reported that coagulation regulation, complement activation, and coagulation cascades constituted the most significantly upregulated biological processes associated with myopia induction in the far-peripheral retina of rabbits (43). The fibrinogen gamma chain may increase in response to elevated retinal vascular permeability induced by ischemia or inflammation. Myopia is strongly correlated with inflammation, with inflammation factors upregulated in myopic eyes and downregulated upon treatment with atropine (44). We also noticed the down-regulation of nitric oxide synthases (NOS) in spontaneously myopic retina. NOS is a critical enzyme catalyzing the production of nitric oxide (NO), which inhibits FDM dose-dependently in chickens and mediates the inhibition of myopia by atropine (45). The NO synthase inhibitor can also alleviate the myopia development in chickens (46) and guinea pigs (47). Overall, the proteomic analysis suggests that spontaneous myopia shares at least some signaling pathways with experimental myopia.

In plasma, the most significantly upregulated proteins were histone H3, histone H2A, and Histone H4, which were enriched in the alcoholism and systemic lupus erythematosus pathway according to KEGG analysis. Proteomic analysis revealed histones H4 and H3 as the most prominently upregulated proteins in RPE-derived exosomes from tree shrews with FDM (48). We also observed upregulation of the VEGF (vascular endothelial growth factor) signaling pathway, with heat shock protein beta 1 (HSPB1) being the most significantly enriched DEP in this pathway. The HSPB1–VEGF interaction critically regulates the balance between physiological and pathological angiogenesis (49). Given that the guinea pig retina is avascular, this signaling pathway may play a regulatory role in choroidal vascular during ocular development.

## 5 Conclusion

In conclusion, this study provides further insights into spontaneous myopia in guinea pigs, suggesting its potential application as a high myopia model. In addition to evident axial myopia and tessellated fundus, we found spontaneous myopia showed very thin retina, choroid and sclera, but normal ERG amplitudes. Meanwhile, active apoptosis was observed in the ONL layer. Proteomics analysis revealed shared retinal signaling pathways between spontaneous and experimental myopia, including upregulated ferroptosis along with complement and coagulation cascades, and downregulated nitric oxide synthases. Considering that spontaneous myopia is not progressive, it might have the potential to be used as a stable high myopia model.

## 6 Limitations

There are several limitations in this study. Firstly, this study was conducted on two-week-old guinea pigs without intervention and longitudinal observation. Protein expression may change with age. Additionally, longitudinal fundus observation could provide insights into whether this form of high myopia progresses to pathological myopia. Secondly, although our Astray DIA analysis identified a panel of DEPs, these findings require independent validation using robust, targeted methods such as Western blot or parallel reaction monitoring (PRM). Subsequent studies should prioritize validating these candidate biomarkers and proceed to functionally verify their roles in spontaneous myopia through perturbation experiments. Thirdly, the choroid and sclera are critical sites where many myopia-related biological processes occur. Since our study provided only retinal and plasma proteomic data, further research is warranted to explore the protein profiles in the choroid and sclera in spontaneous myopia.

## Data Availability

The datasets presented in this study can be found in online repositories. The names of the repository/repositories and accession number(s) can be found in the article/Supplementary material.
