# Supplementary material for: Proteomic profiling of spontaneous myopia in guinea pigs
Source: Front Med (Lausanne). 2026 Mar 19;13:1757831. doi: 10.3389/fmed.2026.1757831 (PMC13043435; doi:10.3389/fmed.2026.1757831)
Supplement: Supplementary file 1 [file Supplementary_file_1.docx]

Supplementary Table 1. Key DEPs identified in retina.

| Protein name | Up/Down | KEGG pathway | Fold change | *P* value |
| --- | --- | --- | --- | --- |
| Fibrinogen gamma chain | Up | Complement and coagulation cascades | 1.56 | 0.03 |
| Alpha-macroglobulin | Up |  | 2.29 | 0.02 |
| ferroxidase | Up | Ferroptosis | 1.33 | 0.01 |
| Solute carrier family 7 member 11 | Up |  | 2.27 | 0.02 |
| EI24 autophagy associated transmembrane protein | Up | p53 signaling pathway | 1.26 | 0.03 |
| Ferroptosis suppressor protein 1 | Up |  | 1.90 | 0.049 |
| Arylamine N_acetyltransferase | Down | Caffeine metabolism | 0.17 | 0.005 |
| Aryl hydrocarbon receptor nuclear translocator like | Down | Circadian rhythm | 0.77 | 0.04 |
| non_specific serine_threonine protein kinase | Down |  | 0.77 | 0.04 |
| Nitric oxide synthase | Down | Phagosome | 0.82 | 0.02 |
| Dynein cytoplasmic 1 intermediate chain 1 | Down |  | 0.73 | 0.04 |
| Syntaxin_18 | Down |  | 0.81 | 0.005 |

*Up/Down: Spontaneously myopic guinea pigs versus hyperopic guinea pigs.

Supplementary Table 2. Key DEPs identified in plasma.

| Protein name | Up/Down | KEGG pathway | Fold change | *P* value |
| --- | --- | --- | --- | --- |
| Histone H3 | Up | Alcoholism | 5.70 | 0.004 |
| Histone H2A | Up |  | 2.26 | 0.02 |
| Histone H4 | Up |  | 3.78 | 0.01 |
| phosphopyruvate hydratase | Up | RNA degradation | 1.81 | 0.02 |
| Stress_70 protein_ mitochondrial | Up |  | 2.27 | 0.01 |
| Heat shock protein beta_1 | Up | VEGF signaling pathway | 1.80 | 0.02 |

*Up/Down: Spontaneously myopic guinea pigs versus hyperopic guinea pigs.
